# Low-Cost Wireless Temperature Measurement: Design, Manufacture, and Testing of a PCB-Based Wireless Passive Temperature Sensor

**DOI:** 10.3390/s18020532

**Published:** 2018-02-10

**Authors:** Dan Yan, Yong Yang, Yingping Hong, Ting Liang, Zong Yao, Xiaoyong Chen, Jijun Xiong

**Affiliations:** 1 Key Laboratory of Instrumentation Science & Dynamic Measurement, Ministry of Education, North University of China, Taiyuan 030051, China; b1506004@st.nuc.edu.cn (D.Y.); hongyingping@nuc.edu.cn (Y.H.); liangting@nuc.edu.cn (T.L.); 2 Science and Technology on Electronic Test & Measurement Laboratory, North University of China, Taiyuan 030051, China; 3 Taiyuan Research Institute Co., Ltd., China Coal Technology and Engineering Group Corporation, Taiyuan 030006, China; s1606077@st.nuc.edu.cn; 4 North Automatic Control Technology Research Institute, Taiyuan 030051, China; b1406004@st.nuc.edu.cn; 5 National Demonstration Center for Experimental Chemical Engineering Comprehensive Education, North University of China, Taiyuan 030051, China

**Keywords:** high- and low-temperature measurement, passive wireless temperature sensor, dielectric constant, PCB substrate

## Abstract

Low-cost wireless temperature measurement has significant value in the food industry, logistics, agriculture, portable medical equipment, intelligent wireless health monitoring, and many areas in everyday life. A wireless passive temperature sensor based on PCB (Printed Circuit Board) materials is reported in this paper. The advantages of the sensor include simple mechanical structure, convenient processing, low-cost, and easiness in integration. The temperature-sensitive structure of the sensor is a dielectric-loaded resonant cavity, consisting of the PCB substrate. The sensitive structure also integrates a patch antenna for the transmission of temperature signals. The temperature sensing mechanism of the sensor is the dielectric constant of the PCB substrate changes with temperature, which causes the resonant frequency variation of the resonator. Then the temperature can be measured by detecting the changes in the sensor’s working frequency. The PCB-based wireless passive temperature sensor prototype is prepared through theoretical design, parameter analysis, software simulation, and experimental testing. The high- and low-temperature sensing performance of the sensor is tested, respectively. The resonant frequency decreases from 2.434 GHz to 2.379 GHz as the temperature increases from −40 °C to 125 °C. The fitting curve proves that the experimental data have good linearity. Three repetitive tests proved that the sensor possess well repeatability. The average sensitivity is 347.45 KHz/°C℃ from repetitive measurements conducted three times. This study demonstrates the feasibility of the PCB-based wireless passive sensor, which provides a low-cost temperature sensing solution for everyday life, modern agriculture, thriving intelligent health devices, and so on, and also enriches PCB product lines and applications.

## 1. Introduction

In various fields such as light industry, modern agriculture, medical care, and even in day-to-day life, there is a need for low-cost temperature monitoring systems (sensors), which are capable of easy integration, taking measurements in low temperature ranges, wireless operation, and in situ temperature monitoring, for the real-time awareness of production safety, standard operation, and personal health monitoring, and so on [1]. For instance, in greenhouses, it is particularly useful for real-time temperature monitoring in order to adjust the plant’s growth status and to achieve accurate farming. Monitoring the ambient temperature in pig pens, poultry houses, and so on, is important for achieving fine control over the health and reproduction of animals. During the storage and transportation of food, the temperature of vegetables, fruits, and meat needs to be monitored in order to reduce the probability of spoilage [2]. Some of the common uses in day-to-day life include monitoring the temperature of car tanks to prevent overheating and dry burning, and the production of intelligent and portable medical monitoring equipment for the real-time monitoring of human body temperature in order to provide 24 h care to the frail elderly, infants, and patients. 

The existing temperature sensing systems (temperature sensors) that are widely used include the thermocouple-type, thermal resistance-type, PN junction (Positive-Negative junction) type, and other lead-type temperature sensors. For instance, thermocouples are used to measure the temperature in small-scale reactors [3], to measure surface temperatures [4], and to measure the temperature between the hot and cold sides of vehicular heat exchangers [5]. In thermal resistance type sensors, the temperature is monitored and used either directly or indirectly [6], and platinum thermal resistance is the most commonly used type in industrial production [7]. The PN junction principle is used to measure the temperature in the literature [8,9]. These sensors achieve temperature sensing and contribute to the measurement of temperature parameters. However, due to the use of a separate temperature sensor and wire connection, there is an increase in the manufacturing cost and interference in measuring temperature, along with an increase in the complexity and difficulty of temperature measurement, system integration, and packaging. In lead-type temperature sensors, the presence of wires makes it not conducive for the temperature monitoring and measurement of moving entities [10].

Surface acoustic wave (SAW), inductive-capacitive (LC), and microwave wireless temperature sensors can avoid the problems of wire connection packaging and achieve wireless communication. Reindl et al. measured temperature with a SAW temperature sensor, the range was 200 °C [11]. Francois et al. successfully used SAW to measure temperatures of above 500 °C [12]. An LC temperature sensor consisting of a spiral inductor and an interdigitated capacitor, connected in parallel, was fabricated by Milan et al. [13]. The LC sensor proposed by Ren et al. can measure the temperature and humidity of the environment simultaneously [14]. Tan et al. reported that an LC resonator was used to measure temperatures of up to 700 °C [15]. These wireless temperature sensors have no lead connection; however, production and integration problems still exist due to independent temperature sensitive structure and materials which are frequently heterogeneous with IC substrate and functional materials, especially PCB based IC circuits.

For this reason, Ahn et al. (from South Korea) proposed a PCB temperature sensor, which is easy to be fabricated following PCB electrical circuit processes and integrated with the PCB circuits, which uses only the copper foil on the polyimide as a thermal resistance for temperature sensing. In essence, it is still a lead-type temperature sensor and is not conducive for wireless communication [16]. Sanders et al. used PCB material to measure temperature. The substrate material is RO3006 and testing range of temperature is 40–100 °C [17]. However, a transmission line and an SMA adapter were required, it did not really implement wireless transmission of signals. Xianwei Shi’s research team presented a passive temperature sensing antenna which achieved the sensitivity larger than 4.00 MHz/°C in a temperature range from 30 °C to 50 °C [18]. The function of temperature measurement is realized by fabricate and assemble a bimetal strip coil, a dipole antenna and a back cavity together, so the production processes are complicated. Stephan et al. designed a 12.5 GHz near-field focused microstrip antenna array to sense the temperature at the range of 20 °C–140 °C [19]. However, the sensor size is 185 mm × 185 mm × 2 mm; it is too large for integration with other devices and realize miniaturization. Yang et al. illustrated a passive and wireless slotted patch antenna with temperature sensation at the working frequency of 900 MHz [20]. However, the testing temperature was only 17–70 °C. 

We proposed a PCB-based, low-cost wireless passive temperature sensor, avoiding the issues mentioned above. We designed and manufactured its prototype structure, confirmed the feasibility of this sensor, and realize the wireless passive way to transmit signals. The sensor was tested regarding its high- and low-temperature sensing ability in the range of −40 to 125 °C. The sensor is made using the PCB material and has a size of 56.2 mm × 70 mm × 1.6 mm, therefore, it has advantages, such as low-cost and easy integration with the PCB circuit, which is suitable for the light industry-oriented temperature systems [21,22,23]. The metal adheres to the upper and lower surfaces of the dielectric substrate, so the manufacturing process of the sensor is simple and does not need any additional devices. At the same time, the sensor is small and light weight, making it highly suitable for daily care applications, logistics industry, and various other temperature systems.

## 2. Measuring Principle 

**Figure 1:**
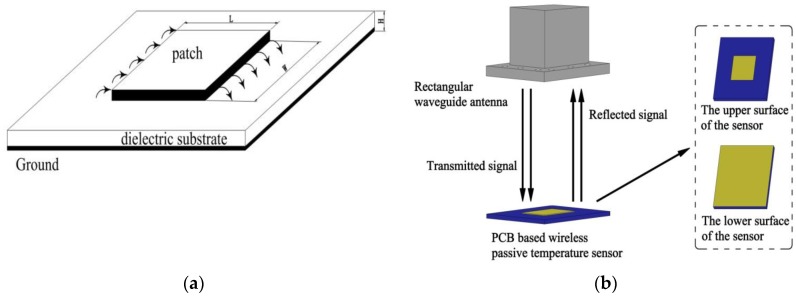
Schematic diagram of the measurement: (**a**) sensor structure and field distribution; and (**b**) signal transmission.

The proposed temperature sensor is a microwave patch antenna type sensor, which contains a dielectric substrate and a patch antenna integrated on it. The upper and the lower surface of the dielectric substrate are covered with metal, which form a patch and metal ground, respectively. The electric field in the dielectric substrate is uniformly distributed in the thickness direction. The field has no change along the width of the patch and only along the length. The radiation field is generated by the fringing fields at the two open ends of the patch in the length direction, as shown Figure 1a. The vertical electric field components at the open end cancel each other in the space and the electric field of the horizontal components are in the same direction. Therefore, the radiation field is mainly generated by the horizontal component field.

The radiation patch antenna is able to receive and send electromagnetic waves to achieve the wireless transmission of the temperature signal. Figure 1b shows the measurement principle of the wireless passive temperature sensor with PCB substrate. The rectangular waveguide antenna (RWA) operates as an interrogation antenna. It sends a sweep signal of a certain bandwidth to the temperature sensor, and the components of the swept signal, which are similar to the center frequency of the sensor, will enter the sensor and form a standing wave of the corresponding frequency. As the sensor is not an ideal resonant cavity, the standing wave will be gradually dissipated over time. The remaining frequency components are reflected back and are received by the RWA. Therefore, the resonant frequency of the sensor can be identified by recording the reflected frequency spectrum received by the RWA.

The resonant frequency of the sensor changes when there is a change in the ambient temperature. There are two main factors that affecting the resonant frequency [17,24]. One factor is metal thermal expansion as the temperature changing. Therefore, the effective length of the patch becomes longer and the resonant frequency decreases. The expression as follows:

(1) $$\frac{\delta f}{f_{r}}=-\frac{\delta L}{L}=-\alpha_{d}\delta T$$

where δf is change in the resonant frequency; $f_{r}$ is the resonant frequency of the sensor; δL is change in effective resonant dimension; *L* is length of metal patch; $\alpha_{d}$ is thermal expansion coefficient; δT is temperature change with unit °C.

Another factor is that the dielectric constant of the substrate material changes with temperature. The dielectric constant of the PCB (FR4) substrate material increases with an increase in temperature [25,26]. Since the resonant frequency of the sensor is inversely proportional to the square root of the dielectric constant, the resonant frequency will decrease. The expression as follows:

(2) $$\frac{\delta f}{f_{r}}=-\frac{1}{2}\frac{\delta \varepsilon_{r}}{\varepsilon_{r}}=-\frac{1}{2}\alpha_{\varepsilon }\delta T$$

where $\delta \varepsilon_{r}$ is change in dielectric constant; $\alpha_{\varepsilon }$ is thermal coefficient of dielectric constant.

Therefore, the relationship between the change in resonance frequency (δf) and the temperature variation (δT) is established, the expression is:

(3) $$\frac{\delta f}{f_{r}}=-\frac{1}{2}\frac{\delta \varepsilon_{r}}{\varepsilon_{r}}-\frac{\delta L}{L}=\left(-\frac{1}{2}\alpha_{\varepsilon }-\alpha_{d}\right)\delta T$$

The resonant frequency is linear with temperature from the above equation.

## 3. Design and Fabrication

### 3.1. Sensor Design 

In this study, a wireless passive temperature sensor based on a PCB substrate is designed. The temperature sensor operates in the ${\mathrm{TM}}_{01}$ mode, and the resonant frequency is calculated as follows:

(4) $$f_{r}=\frac{c_{0}}{2L_{e}\sqrt{\varepsilon_{e}}}$$

where $c_{0}$ is the speed of light in vacuum; $L_{e}$ is the actual length of the radiation patch while considering the fringing field effect [27]; and $\varepsilon_{e}$ is the effective permittivity of the substrate.

The length of the radiation patch is calculated as follows:

(5) $$L_{e}=\frac{c_{0}}{2f_{r}\sqrt{\varepsilon_{e}}}-2\Delta L$$

where $f_{r}$ is the resonant frequency of the temperature sensor ($f_{r}=2.45\;\mathrm{GHz}$ at room temperature); and Δ*L* is the equivalent radiation gap length, which can be calculated by:

(6) $$∆L=0.412\;h\frac{\left(\varepsilon_{e}+0.3\right)\left(\frac{w}{h}+0.264\right)}{\left(\varepsilon_{e}-0.258\right)\left(\frac{w}{h}+0.8\right)}$$

where *h* (= 1.6 mm) is the thickness of the substrate; and *W* is the width of the radiation patch, which is calculated as follows:

(7) $$W=\frac{c_{0}}{2f_{r}}{\left(\frac{\varepsilon_{r}+1}{2}\right)}^{-\frac{1}{2}}$$

where $\varepsilon_{r}$ is the dielectric constant of the substrate; and $\varepsilon_{r}=4.4$ at room temperature [28,29].

The effective permittivity $\varepsilon_{e}$ is calculated as follows:

(8) $$\varepsilon_{e}=\frac{\varepsilon_{r}+1}{2}+\frac{\varepsilon_{r}-1}{2}{\left(1+12\frac{h}{w}\right)}^{-\frac{1}{2}}$$

Therefore, the theoretical calculation of the sensor is: L=28.83 mm, W=37.26 mm.

**Figure 2:**
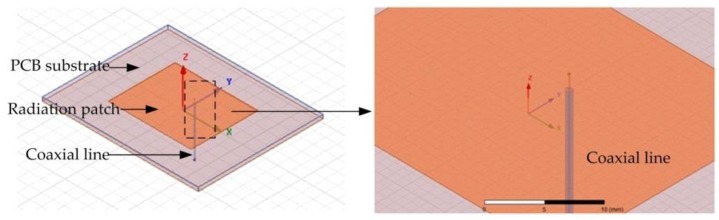
The schematic of the temperature sensor prototype in HFSS.

**Figure 3:**
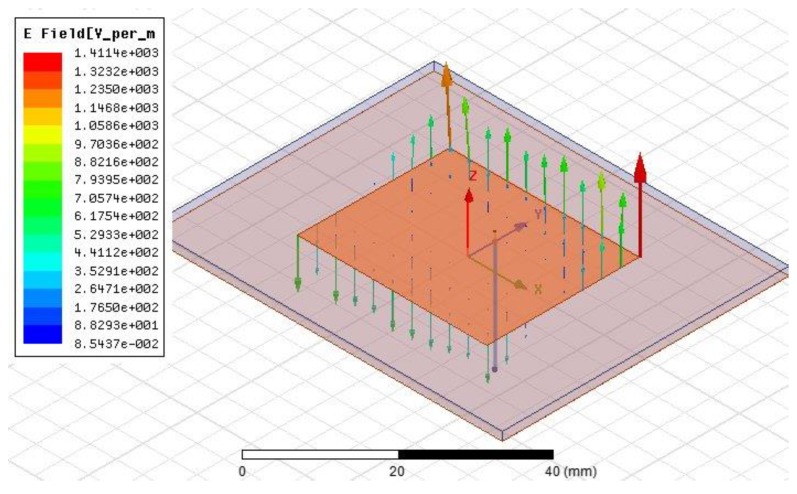
Electric field distribution of the sensor.

**Figure 4:**
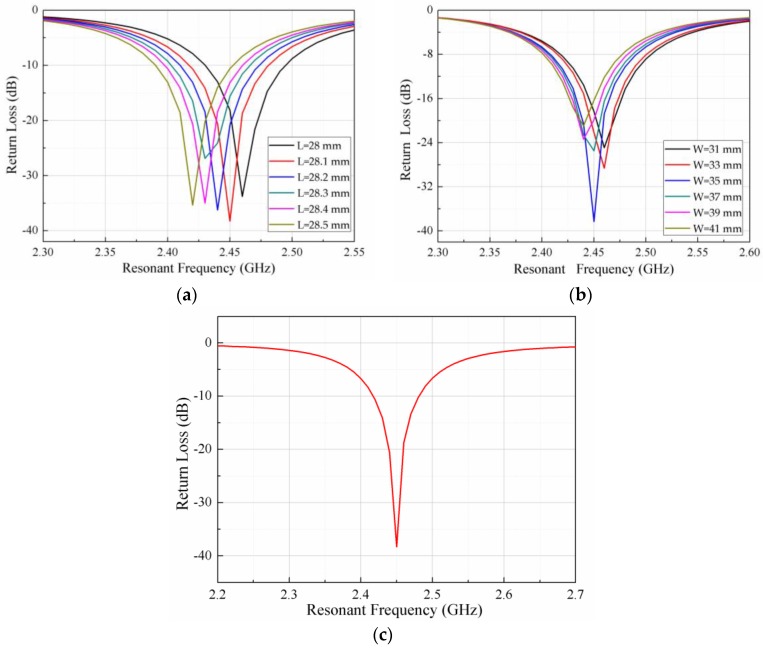
HFSS simulation results: (**a**) the effect of radiation patch length on the resonant frequency of the sensor; (**b**) the effect of radiation patch width on the resonant frequency of the sensor; and (**c**) the resonant frequency of the sensor.

To improve the radiation efficiency of the sensor and to reduce the transmission loss, the sensor model is simulated and analyzed in the HFSS (High Frequency Structure Simulator) 15.0 version software (Southpointe 2600 ANSYS Drive, HFSS, ANSYS, Inc., Canonsburg, PA, USA). A coaxial cable is inserted into the PCB-based temperature sensor to excite the sensor and form a weak coupling relationship with the sensor, as shown in Figure 2. The coaxial line is used only for the simulation analysis and will not appear in the actual sensor. To illustrate the working principle of the temperature sensor further, we refer to the electric field distribution as shown in Figure 3. It can be seen from the figure that the electric field intensity is the strongest at the edge of the radiation patch. Thus, it can be considered that the radiation is caused by the edge of the open side of the radiation patch. The horizontal components of the electric field at the two open ends can be considered to be equivalent to two gaps, and are consistent with the theoretical analysis and calculation. The size of the radiation patch and the resonant frequency of the sensor are simulated and optimized, as shown in Figure 4. The optimized curves for the length and width of the radiation patch are shown in Figure 4a,b, respectively. It can be seen from the figures that the resonant frequency of the sensor decreases when the length of the radiation patch L increases; the resonant frequency of the sensor is 2.45 GHz when L = 28.1 mm. The change in width of the radiation patch W has little effect on the resonant frequency of the sensor. When W = 35 mm, the return loss of the curve is the smallest, and the radiation effect of the sensor is at its best. The resonant frequency of the sensor, as optimized by the HFSS software is 2.45 GHz, which is the trough of the curve in Figure 4c, where the return loss is −38.23 dB.

### 3.2. Sensor Fabrication

There are many PCB substrate materials suitable for microwave frequency bands, such as PTFE (polytetrafluoroethylene) substrate, RO4003, epoxy glass (FR4) et al. PTFE is a kind of high performance thermoplastic polymer and can be stable work in a wide frequency range. It has a low dielectric constant $\varepsilon_{r}=2.1$ [30]. However, the relationship between substrate size and dielectric constant is inversely proportional [31]:

(9) $$l\propto \lambda =\frac{c}{f\sqrt{\varepsilon_{r}\mu_{r}}}$$

as a result the overall size of the substrate becomes large, making it impossible to achieve a real miniaturization. In additional, the PTFE substrates require special processing techniques for fabrication, which are more expensive. RO4003 laminate is a high frequency performance and low cost substrate material. It has low dielectric loss and high radiation efficiency. However, the thermal coefficient of dielectric constant is only 40 ppm/°C, which is too low for the sensing temperature [32]. FR4 substrate is one of PCB dielectric materials which is widely used for microwave circuits and antennas. The dielectric constant and loss tangent of the substrate are $\varepsilon_{r}=4.4$ and tanδ=0.02 respectively, while the thermal coefficient of dielectric constant is 160 ppm/°C. The thermal expansion coefficient of FR4 material is 16 ppm/°C, almost matched to the thermal expansion coefficient of the metallic copper (17 ppm/°C). Therefore, it is not prone to delamination and shedding phenomenon at high temperature. FR4 substrate also has excellent mechanical properties, manufacturing process simplicity, and the advantage of low cost [33]. Thus, the FR4 material is selected as the substrate in this paper.

As the high-frequency electromagnetic wave (GHz) propagates in the conductor, the current excited by the conductor only exists in the surface area of the conductor, the phenomenon called skin effect. The electric field strength decays exponentially when electromagnetic waves propagate inside the conductor. The depth decays to 1e of conductor surface, this depth value is called skin depth (δ) [34]. The formula is:

(10) $$\delta =\frac{1}{\sqrt{\pi f\mu_{1}\sigma_{1}}}$$

where, δ—skin depth, unit is m; *f*—resonant frequency, unit is Hz; $\mu_{1}$—magnetic permeability, unit is $H\cdot m^{-1}$; $\sigma_{1}$—conductivity, unit is $S\cdot m^{-1}$.

Copper is adopted as a conductor in this paper, the parameters ($\mu_{1}=4\pi \times 10^{-7}\;H\cdot m^{-1}$; $\sigma_{1}=5.8\times 10^{7}\;S\cdot m^{-1}$) were substituted into the above formula to get the expression as following:

(11) $$\delta =\frac{0.066}{\sqrt{f}}$$

**Figure 5:**
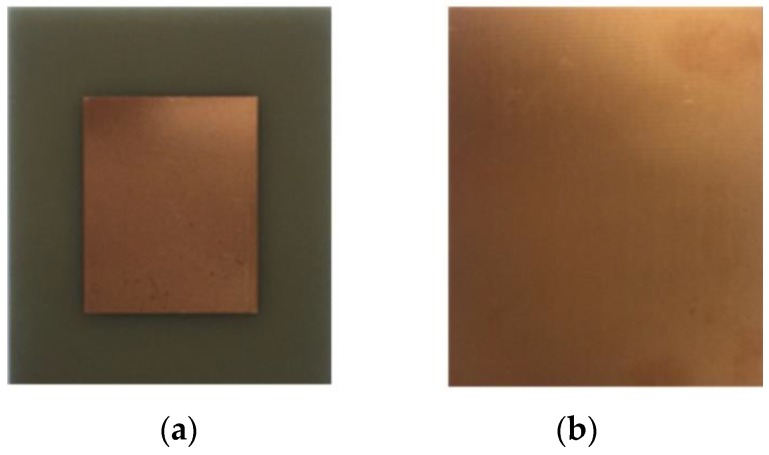
Sensor fabricated: (**a**) radiation patch on the upper surface of the sensor; and (**b**) metallic ground on the lower surface of the sensor.

The skin depth was calculated to be 1.33 μm for copper. In order to eliminate the skin effect, the thickness of the metal layer should be 3–5 times of the skin depth, so the thickness of the copper is about 4.3 μm [35]. The size of the substrate is 56.2 mm × 70 mm × 1.6 mm, which obtained from optimization of HFSS software. The sensor fabricated is shown in Figure 5.

## 4. Testing and Discussion

A high-temperature and a low-temperature test system are set up to test the high-temperature and low-temperature performances of the PCB-based temperature sensor, respectively.

### 4.1. High-Temperature Test 

**Figure 6:**
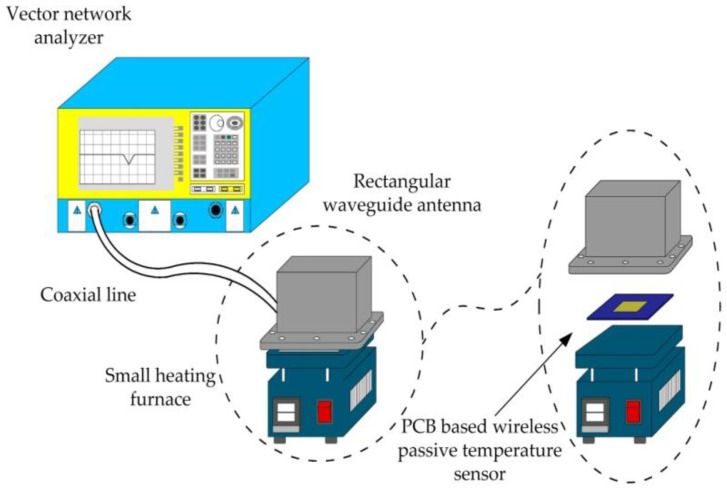
High-temperature testing system.

The high-temperature testing system consists of three sectors: a vector network analyzer (N5224A, 10 MHz–43.5 GHz, Agilent, Santa Clara, CA, USA), small heating furnace, and RWA. The system is shown in Figure 6. The small heating furnace is used to heat the sensor. The RWA is used to transmit and receive signals and is connected with the vector network analyzer by a coaxial line. The vector network analyzer is used to monitor and display the scattering signal of the sensor, which is represented in the form of the logarithmic amplitude of the S11.The trough of the S11 curve is the resonant frequency of the sensor at the measuring temperature. The testing system can detect the frequency change of the sensor with temperature in real-time.

The temperature sensor is placed in a small heating furnace, and the RWA is located 50 mm above the temperature sensor. The vector network analyzer is calibrated and the sweep frequency is set at a range of 2–3 GHz. The furnace heats the PCB-based temperature sensor at a rate of 2 °C/min. The temperature is increased by 20 °C and hold for 5 min, the experimental data is recorded during this period. The testing range is 25–125 °C.

The higher end temperature (125 °C) is determined by the glass transition temperature of FR4. The glass transition temperature (Tg) is the critical temperature at which the coefficient of thermal expansion suddenly changing from a lower value to a higher value [36]. The glass transition temperature of FR4 is about 125–135 °C [37,38]. Above the glass transition temperature, the mechanical properties of substrate material degrade due to the softening of the resin and large discontinuous changes in the thermal expansion coefficient which even to appear delamination in the board [39]. Therefore, the temperature was measured from room temperature to 125 °C.

**Figure 7:**
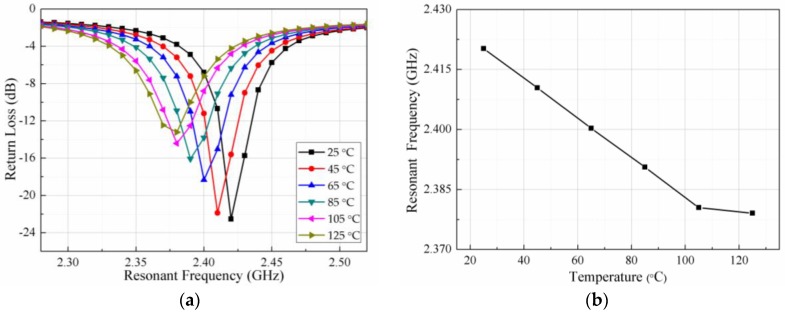
Relationship between temperature and the resonant frequency during high temperature process: (**a**) return loss vs. resonant frequency at various temperatures; and (**b**) extracted resonant frequency vs. temperatures.

The relationship between the resonant frequency and the temperature of the sensor during the temperature rise is shown in Figure 7a. The dielectric constant of the PCB substrate increases slowly with an increase in temperature, resulting in the resonant frequency of the sensor to decrease gradually, as seen from the trough of the S11 curve moving gradually to the left. The impedance matching deteriorates due to the dielectric loss and the conductor loss becoming large when the temperature rises. Therefore, the return loss of the curve becomes larger. To illustrate the change in resonant frequency with the temperature clearly, the trough points of the curve in Figure 7a are extracted and plotted in Figure 7b. As can be seen from Figure 7b, the actual measured value is 2.42 GHz at room temperature, while the resonant frequency simulation value of 2.45 GHz. This difference is caused by machining errors. The difference between the measured frequency and the simulated frequency is only 30 MHz (<40 MHz), and this proves that the measured and the simulated value are in good agreement [40]. The resonant frequency of the sensor is reduced from 2.42 GHz to 2.379 GHz when the temperature rises from room temperature (25 °C) to 125 °C. There is a slight deviation of the resonant frequency at 125 °C, the possible reason is the dielectric constant of substrate material changing abruptly near the glass transition temperature.

### 4.2. Low-Temperature Test

**Figure 8:**
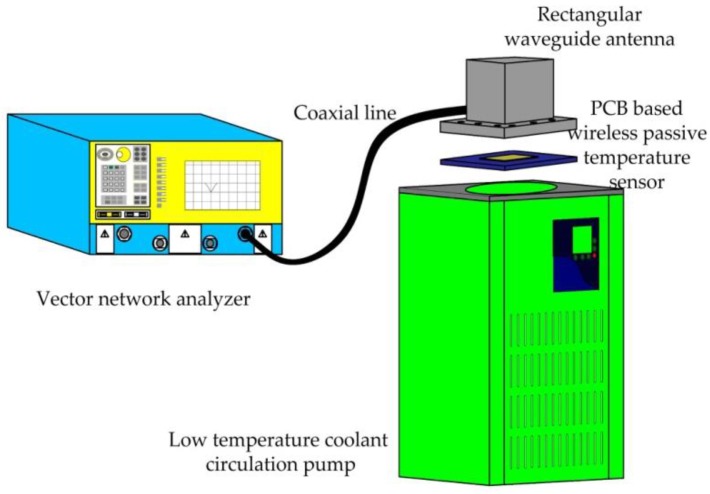
Low-temperature testing system.

The low-temperature testing system contains of three apparatus: vector network analyzer, rectangular waveguide antenna, and low temperature coolant circulation pump (DLSB-5L/40, Gongyi City Yuhua Instrument Co., Ltd., Gongyi, China). The system diagram is shown sketchily in Figure 8. The vector network analyzer is connected to the rectangular waveguide antenna by a coaxial line for the transmission and reception of the signal, and the S11 curve of the return signal is displayed on the screen of the vector network analyzer. The role of the low temperature coolant circulation pump is to cool the PCB-based temperature sensor.

**Figure 9:**
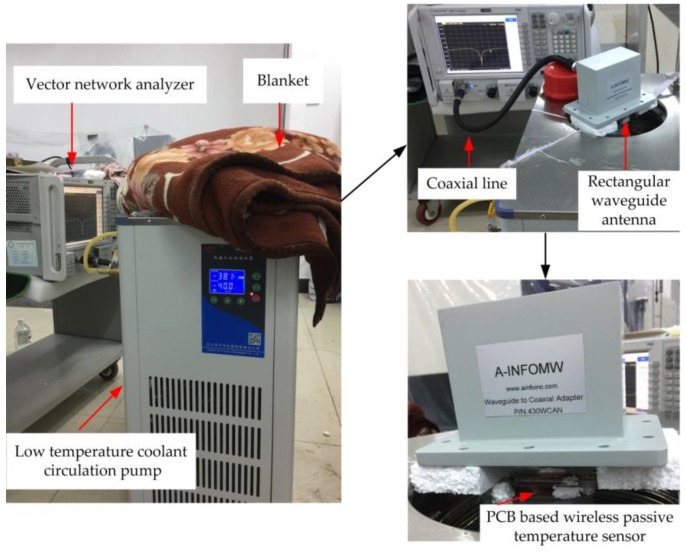
Actual low-temperature testing system.

Five liters of the coolant is poured into the low-temperature coolant circulation pump. The coolant contains a certain proportion of ethylene glycol aqueous solution. The PCB-based temperature sensor is located on the upper surface of the coolant. The rectangular waveguide antenna is about 50 mm above the sensor and supported by insulating foam. The vector network analyzer is set to a sweep signal range of 2.2–2.6 GHz. The coaxial line is used to connect the rectangular waveguide antenna to the vector network analyzer for monitoring the resonant frequency of the sensor. When the power button is turned on, the circulating pump starts to cool down and the real-time temperature is read through the temperature screen. Each time the temperature is reduced by 10 °C and hold for 5 min, the experimental data is recorded. To ensure stability of the temperature, so that the accuracy of the measurement is improved, a multi-layer blanket is used to cover the rectangular waveguide antenna and sensor to prevent heat conducting between the rectangular waveguide antenna and sensor and outside environment. The actual low-temperature testing system is shown in Figure 9.

**Figure 10:**
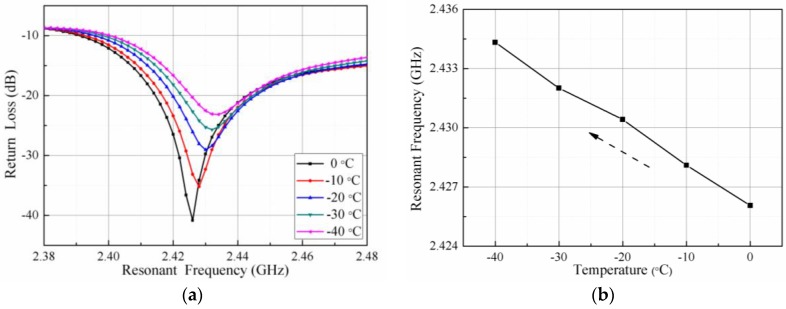
Relationship between the temperature and resonant frequency in a low-temperature testing: (**a**) return loss vs. resonant frequency at various temperatures; and (**b**) extracted resonant frequency vs. temperatures.

Due to the limited temperature range of the coolant circulation pump itself, the temperature of the low temperature test can only be 0 to −40 °C. The relationship between the resonant frequency and the temperature of the sensor during the cooling process is shown in Figure 10a. The trough of the curve gradually shifts to the right when the temperature decreases. From the trough point, namely the resonant frequency of the sensor, it can be seen that the resonant frequency of the sensor increases gradually. The trough points of the curve shown in Figure 10a are extracted and plotted as shown in Figure 10b. The resonant frequency of the sensor changes from 2.426 to 2.434 GHz when the temperature falls from 0 °C down to −40 °C.

**Figure 11:**
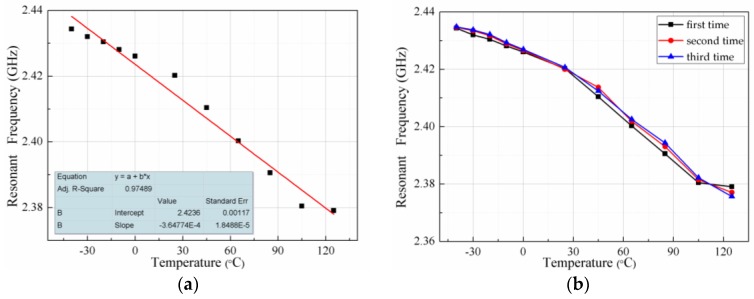
Relationship between temperature and frequency throughout the testing process: (**a**) curve fitting; and (**b**) repeatability test.

Figure 11a shows the fitting curve of resonant frequency versus temperature for the entire test temperature range (−40 °C to 125 °C). The determination coefficient ($R^{2}=0.97489$) in the fitting curve illustrates that the curve has a good linearity and the experimental data have high reliability compared with the others sensors based on the principle of microwave transmission and LC resonant sensors [13,41,42,43]. To ensure the accuracy of experimental results and verify the repeatability of the sensor, the sensor was tested for three times while maintaining the same test environment. The test results are shown in Figure 11b, the test curve has only one abrupt frequency point at 125 °C, and the repeatability is good compared with the general ceramic dielectric resonator [20]. The average sensitivity of the sensor is $S_{f}=\frac{\Delta f}{\Delta t}=347.45\;\mathrm{KHz}/{}^{\circ}C℃$ according to the measurement results. However, the theoretical value is 156.8 KHz/°C from Equation (3). The measured sensitivity is much larger than the theoretical value. The reason for this phenomenon is that the dielectric constant changes greatly due to the moisture absorbed by the sensor during the test. The dielectric constant and loss tangent vary with operating temperature changes and levels of humidity [44]. The dielectric constant and dielectric loss of PCB materials increase under the effect of humidity condition. The dielectric constant of water molecules is about 80, so even a small amount of absorbed moisture significantly changes the dielectric properties [45]. The resonant frequency of the sensor will also increase dramatically with the change of the dielectric constant. Therefore, the actual measurement sensitivity is higher than the theoretical value.

**Figure 12:**
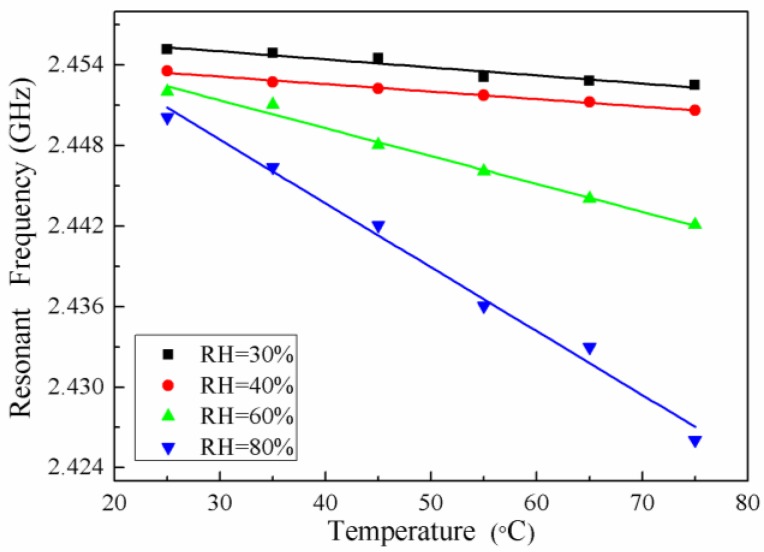
Fitting curve in different humidity levels.

In order to verify whether the humidity has an influence on the sensor, the relationship between the frequency and the temperature is measured under a constant humidity. The humidity value is RH = 30%, RH = 40%, RH = 60% and RH = 80%, respectively, the temperature range is 25–75 °C, and the test curve is shown in Figure 12.

It can be seen from Figure 12 that the resonant frequency of the sensor decreases with increasing of temperature when humidity is constant, and the relationship between them changes linearly. The resonant frequency decreases with increasing humidity when the temperature is constant. The variation resonant frequency of the sensor is greater as the higher of the humidity value. Therefore, it is proved that the sensor is affected by humidity and consistent with theoretical analysis.

## 5. Conclusions

This paper presented a PCB-based wireless passive low-cost temperature sensor. The advantages of this sensor are simple structure, small size, easy processing, convenient integration, and low-cost. The feasibility of applying the PCB material for the measurement of temperature and PCB-based wireless passive sensor are proved through theoretical analysis, simulation, processing, and measurements. The resonant frequency is obtained from 2.434 to 2.379 GHz in the temperature range −40 to 125 °C through the high- and low-temperature tests, respectively. The linear fitting curve has good linearity and high reliability of the experimental data. Three repeated tests prove the good repeatability. The average sensitivity of the sensor is 347.45 KHz/°C℃. The measured sensitivity is much larger than the theoretical value. In our future work, we will improve the measurement range of the sensor by ameliorating the test system and designing a high-gain broadband interrogation antenna based on the PCB substrate.
